# Positive emotional cues at work: how customer empowering behavior promotes proactive customer service via psychological safety

**DOI:** 10.3389/fpsyg.2026.1734905

**Published:** 2026-02-16

**Authors:** Wei Chen, Kai Yao, Zhaoying Fan

**Affiliations:** 1 Rattana Bundit University, Bangkok, Thailand; 2 Xi'an Jiaotong University, Xi'an, China

**Keywords:** affective events, customer empowering behavior, positive emotional cues, proactive customer service, psychological safety, regulatory focus

## Abstract

Drawing on affective events theory and social exchange perspectives, we conceptualize customer empowering behavior (CEB) as a positive emotional cue that heightens employees’ psychological safety, thereby promoting approach-oriented proactive customer service. We further propose that regulatory focus (promotion vs. prevention) shapes these emotion-to-action pathways. We tested a moderated mediation model in a three-wave, multi-source field study of frontline service employees in Thailand (Time 1: CEB and regulatory focus; Time 2: psychological safety; Time 3: supervisor-rated proactive customer service; *N* = 236 employees nested within 32 supervisors). Hierarchical regressions and bootstrapped conditional-process analyses showed that CEB positively predicted proactive customer service and that psychological safety partially mediated this relationship. Moreover, promotion focus strengthened, whereas prevention focus weakened, the effect of CEB on psychological safety, yielding a stronger indirect effect of CEB on proactive service via psychological safety under high promotion and a weaker one under high prevention focus. These findings position CEB as a positive affective event in service encounters, identify psychological safety as the emotional pathway through which empowering cues translate into discretionary effort, and offer actionable implications for shaping customer interactions and motivational climates that enhance employees’ positive emotional experiences at work.

## Introduction

For many years, slogans like “customer first” and “customer is king” have reflected the dominant role customers play in service relationships and have been widely embraced as guiding principles in service management (Prahalad and Ramaswamy, 2004). This underscores the power dynamic in the interactions between employees and customers, where customers hold a dominant position. When customers interact with employees and transfer some or all of their decision-making authority to them, allowing employees to engage in their consumption decisions, employees perceive this as customer empowerment. This phenomenon, known as customer empowering behavior, occurs when employees feel authorized by customers to exercise independent decision-making to meet service expectations (Dong et al., 2015; Tuan et al., 2019). Customer empowering behavior is typically manifested in scenarios such as when customers request that waiters assist in selecting food, allow drivers to choose a route, permit barbers to decide on a hairstyle, or ask travel agencies for recommendations. Drawing on Affective Events Theory, employees’ psychological safety at work, which in frontline service contexts can be shaped by customer–employee interactions—an affective assessment of interpersonal risk—which, in turn, motivates employees to engage in proactive customer service (Houlfort et al., 2015; Conger and Kanungo, 1988; Liu and Yang, 2017).

Although both academia and industry have gradually recognized that customer empowering behavior is widely present in the contemporary service industry and significantly influences frontline employees’ attitudes and behaviors during service delivery, scholarly attention to this topic remains limited. Existing research has only provided preliminary discussions on customer empowering behavior, with few empirical investigations exploring its mechanisms and outcomes in depth (Dong et al., 2015; Tuan et al., 2019; Öksüz, 2021). A review of the extant literature indicates that most studies examine the psychological and behavioral consequences of employees’ perceptions of customer empowering behavior, primarily from the employees’ perspective (Dong et al., 2015; Tuan et al., 2019; Houlfort et al., 2015). These studies generally conceptualize customer empowering behavior as a form of power transfer that motivates employees, as reflected in the prevailing definitions of the construct (Dong et al., 2015; Tuan et al., 2019; Öksüz, 2021; Yu et al., 2018).

The positive effects of customer empowering behavior on employees may have certain limits. From the perspective of social exchange theory (Houlfort et al., 2015), customer empowering behavior, as a form of power transfer between customers and employees, can provide employees with greater work autonomy, enhance their psychological safety, and motivate them to fully engage their initiative, ultimately leading to improved service performance. However, it is also important to acknowledge that not all employees will perceive this transfer of power as a positive signal. Being empowered by customers often entails assuming greater responsibility, which can induce fear of causing customer dissatisfaction and facing negative consequences, such as complaints or negative reviews, due to their “self-assertion.” This fear may reduce employees’ psychological safety. In such cases, the employee’s regulatory focus—whether promotion or prevention—becomes especially significant. Individuals with different regulatory focuses have distinct perceptions and responses to customer empowering behavior, which may either strengthen or weaken its positive effects (Higgins, 1997; Higgins, 1998; Zhou et al., 2023).

To address this, the current study, grounded in social exchange theory and further integrated with regulatory focus theory, posits that employees’ regulatory focus (promotion vs. prevention) is a crucial moderating factor that determines the impact of customer empowering behavior. For frontline service employees with a promotion focus, who are oriented toward achieving positive outcomes, customer empowering behavior is often viewed as a motivating force. This perception enhances their psychological safety and drives their proactive customer service behavior, as they seek to repay customers’ affirmation and trust (Higgins, 1997; Higgins, 1998). In contrast, frontline employees with a prevention focus, who are more attuned to potential negative outcomes, may interpret customer empowering behavior as a source of risk. As a result, the positive effects of customer empowering behavior may be diminished, reducing its influence on employees’ psychological safety and proactive service behaviors (Lee et al., 2023).

Building on the theoretical framework outlined above, this study incorporates employees’ psychological safety and regulatory focus as mediating and moderating variables to explore the mechanisms and consequences of customer empowering behavior’s impact on employees’ proactive service behavior. The findings of this study not only advance theoretical understanding of how customer empowering behavior influences employee psychology and behavior, but also offer practical policy recommendations for service organizations and frontline employees to effectively embrace customer empowering behavior and maximize its positive effects (Taylor et al., 2012).

## Literature review and research hypotheses

1 Customer empowering behavior.

As an evolving concept derived from empowerment, customer empowering behavior refers to actions in service settings where customers highlight the importance of employees’ work and grant them a degree of autonomy, allowing employees to independently determine how to provide high-quality service based on their own skills and experience (Dong et al., 2015; Tuan et al., 2019). Customer empowering behavior is manifested in several ways during service interactions: (a) emphasizing the significance of employees’ work, meaning that customers acknowledge the importance of the service employees provide; (b) granting employees work autonomy, allowing them to make independent decisions regarding service content and delivery methods; (c) expressing trust in employees’ abilities, indicating belief in their competence to carry out service tasks; and (d) permitting employees to express opinions or offer suggestions, enabling them to fully or partially participate in customer decision-making, which in turn motivates employees to strive for better service performance (Dong et al., 2015; Tuan et al., 2019).

Furthermore, regarding the effects and consequences of customer empowering behavior, Dong et al. (2015) argue that customer empowering behavior can enhance service employees’ desire for success, thereby increasing their likelihood of engaging in service innovation. When employees successfully implement service innovations, they provide customers with an experience that exceeds expectations, leading to higher customer satisfaction. Additionally, when service employees perceive that the leadership style within their organization is empowering, it strengthens their work autonomy and boosts their motivation to deliver high-quality services through independent decision-making, ultimately improving service performance. Moreover, to sustain a competitive advantage in the workplace, employees are increasingly concerned not only with their current job performance but also with their long-term career development. Consequently, customer empowering behavior impacts not only employees’ present work performance but also their future career growth (Dong et al., 2015; Deng et al., 2022).

2 Customer empowering behavior and employee service proactive behavior.

During service encounters, customer empowering behavior enables frontline service employees to make independent decisions without interference from customers, fostering a sense of capability, confidence, and freedom to apply their professional knowledge and work experience to complete service tasks. This empowerment enhances employees’ desire and commitment to achieve high-quality results (Dong et al., 2015). Based on this, the study predicts that customer empowering behavior will significantly enhance the proactive service behaviors of frontline employees.

Customer empowering behaviors, such as showing appreciation or offering autonomy, serve as key emotional cues that employees encounter in service interactions. According to Affective Events Theory (AET), such positive customer behaviors elicit emotional responses from employees, leading to enhanced emotional engagement and proactive behaviors. These emotional reactions reduce employees’ perceived interpersonal risks, motivating them to act more autonomously and proactively in customer service situations (Weiss and Cropanzano, 1996). As a result, employees not only feel more confident but also become more inclined to anticipate and respond to customer needs effectively.

In specific service scenarios, frontline service employees frequently interact with customers and, therefore, require a certain degree of work autonomy to address customers’ diverse and often urgent needs (Griffin et al., 2007). This autonomy, granted by customers, serves as a crucial prerequisite for frontline employees to deliver high-quality service. When employees perceive customer authorization behavior during service delivery, they interpret it as a sign that customers trust their abilities, which in turn boosts their confidence in completing service tasks (Dong et al., 2015). This enhanced confidence encourages employees to take greater initiative in service interactions, fostering more proactive customer service behaviors (Rank et al., 2007). Therefore, this study posits that customer authorization behavior positively influences employees’ proactive customer service behaviors.

*H1*: Customer empowering behavior has a positive effect on employees’ proactive customer service behavior.

3 The mediating role of psychological safety.

Psychological safety refers to employees’ perception of the level of safety they feel when expressing themselves within the organization. It is reflected in employees’ ability to openly express their thoughts at work without fearing suppression by the organization or others, which could negatively impact their image, status, or career development. This sense of psychological safety primarily stems from employees’ belief that the organization or their colleagues respect and trust them, agree with and support their views, and appreciate the content and methods of their expressions (Houlfort et al., 2015). In the service industry, where service content is often non-standardized (Liao and Chuang, 2004), employees must leverage their knowledge, skills, and experience. By expressing themselves to a certain extent, they can effectively complete service delivery to customers (Chen et al., 2023; Liu and Gu, 2018).

In line with prior research, psychological safety in this study refers to employees’ general perception that they can express themselves and take interpersonal risks at work without fear of negative consequences (Liang et al., 2012). Importantly, within frontline service contexts, such perceptions of psychological safety can be shaped not only by interactions with supervisors and coworkers, but also by repeated interactions with customers, who represent salient external social actors in employees’ daily work.

During service delivery, customer empowering behavior reflects the customer’s respect and trust in frontline service employees, providing them with a degree of autonomy and enabling them to make independent decisions regarding how to deliver high-quality service (Dong et al., 2015; Tuan et al., 2019). This, in turn, reduces employees’ perceived interpersonal risk associated with acting autonomously in their work role during the service process, thereby enhancing their psychological safety. With higher psychological safety, employees are more likely to engage in service-oriented behaviors to reciprocate customers’ trust and recognition. Therefore, drawing from social exchange theory, this study proposes that psychological safety serves as a mediator in the relationship between customer empowering behavior and service-oriented behavior.

According to social exchange theory, social interactions are fundamentally based on exchanges, where individuals receive benefits from others and, in turn, provide something in return to maintain fairness and the sustainability of these relationships (Higgins, 1998). In the organizational context, a similar social exchange relationship exists between employees and organizations. When organizations provide benefits such as competitive salaries and promotion opportunities, employees reciprocate by increasing their work enthusiasm and improving work efficiency (Houlfort et al., 2015). This reciprocal exchange also extends to service encounters between employees and customers. When employees perceive that customers affirm and trust them, they are more likely to reciprocate by providing rewards to customers through enhanced service.

According to social exchange theory, trust and affirmation from others are perceived as beneficial rewards in social interactions, prompting individuals to reciprocate (Higgins, 1998). When frontline service employees perceive customer empowering behavior during service encounters, they feel affirmed and trusted in their abilities, which reinforces their sense of the importance of their work. This sense of validation allows employees to focus on delivering high-quality service without fearing customer resentment. Additionally, customer empowering behavior signals the customer’s respect for employees, boosting their self-esteem and preventing feelings of inferiority during interactions with customers. This process enables employees to express themselves more confidently and maintain a high level of psychological safety. In essence, customer empowering behavior fosters employees’ psychological safety, encouraging them to engage more proactively in service delivery.

Research indicates that employees’ psychological safety enhances their motivation to express themselves and encourages the implementation of behaviors that improve work performance (Houlfort et al., 2015). During service encounters, when frontline service employees’ psychological safety is maintained at a high level, they are more likely to utilize their abilities and experiences autonomously and confidently in interactions with customers. This, in turn, fosters more proactive service behaviors, enabling employees to better repay customers’ trust and affirmation. From a psychological perspective, employees’ psychological safety is a critical antecedent that influences their proactive service behaviors toward customers. Employees are only willing to engage in proactive service behaviors when they are certain that their psychological safety is high, in accordance with the principles of social exchange theory.

Beyond the rational reciprocity logic of social exchange, service encounters are also emotional experiences. Drawing on Affective Events Theory, we conceptualize customer empowering behavior (CEB)—which includes customers’ affirmation of the importance of employees’ work, trust in their competence, and the granting of discretion—as a positive emotional cue. These cues prompt employees to perceive the interaction as interpersonally safe, enhancing their psychological safety, which is the affective assessment that one can express and act without fear of interpersonal costs. This elevated psychological safety, in turn, fosters approach-oriented action tendencies, enabling frontline employees to mobilize their knowledge and proactively take initiative on behalf of customers. This emotional pathway helps explain how CEB translates into proactive customer service beyond mere instrumental exchange.

Although psychological safety has traditionally been examined within internal organizational relationships, affective events theory suggests that salient external interactions—such as empowering encounters with customers—can also influence employees’ affective appraisals of interpersonal risk at work.

In summary, this study suggests that customer empowering behavior likely promotes employees’ proactive service behavior by enhancing their psychological safety. Therefore, this study proposes:

*H2*: Psychological safety mediates the relationship between customer empowering behavior and employee proactive customer service behavior.

4 The regulatory role of regulatory focus (Promotion vs. Prevention).

Social exchange theory posits that the process of establishing exchange relationships is fraught with uncertainties and risks. Individuals with different characteristics evaluate these uncertainties and risks in distinct ways (Zhou and Zheng, 2022; Higgins, 1997). As a key individual trait, regulatory focus influences how people perceive social exchange processes and, consequently, shapes their psychological and behavioral responses. According to regulatory focus theory, individuals strive to control or alter their thoughts and reactions to achieve specific goals, a process referred to as self-regulation (Higgins, 1998). Depending on their cognitive styles, individuals develop different regulatory tendencies, leading to significant variations in their self-regulatory approaches.

Regulatory focus theory explains how employees translate empowering cues into action by shaping the emotional significance of customer empowering behavior (CEB). Employees with a promotion focus, who are oriented toward growth and advancement, tend to perceive empowering cues as emotionally rewarding opportunities, thereby enhancing feelings of safety and triggering approach-oriented motivation. In contrast, employees with a prevention focus, who are concerned with security and avoiding loss, may view the same autonomy as a potential risk, leading to heightened vigilance and reduced feelings of safety. As a result, promotion focus strengthens, while prevention focus weakens the relationship between CEB and psychological safety, as well as the downstream indirect effect of CEB on proactive customer service through psychological safety (Qian et al., 2024).

Regulatory focus theory distinguishes between two regulatory styles: promotion focus and prevention focus. The former is closely associated with an individual’s desire for advancement, such as development and growth, while the latter relates to the individual’s need for security, including protection and freedom from harm (Higgins, 1997; Higgins, 1998). Specifically, individuals with a promotion focus are more result-oriented, emphasizing gains, focusing on growth opportunities, and willing to invest effort to achieve their desired goals (Higgins, 1998; Gao et al., 2020). In contrast, individuals with a prevention focus are more safety-oriented, emphasizing the avoidance of losses, focusing on preventing harm, and tending to adopt conservative strategies to avoid mistakes (Higgins, 1997; Das et al., 2020). As such, promotion-focused individuals are more attuned to positive outcomes and are sensitive to positive stimuli, whereas prevention-focused individuals are more concerned with potential negative outcomes and are thus more sensitive to negative stimuli (Higgins, 1998; Zou and Chan, 2019). These two regulatory styles are considered independent and relative concepts, both of which are unified within the broader framework of regulatory focus theory (Higgins, 1998).

In service encounters between frontline service employees and customers, while customer empowering behavior provides employees with a certain degree of autonomy and independent decision-making power during service delivery, it also comes with increased responsibility. The extent to which employees are willing to accept customer empowering behavior and the corresponding impact on their psychological and behavioral responses depend on their regulatory focus (Higgins, 1997). For frontline service employees with a promotion focus, who are more concerned with achieving positive outcomes, they are sensitive to the potential positive results of customer empowering behavior. They tend to view the autonomy and freedom granted by customer empowerment as opportunities for continuous learning and self-improvement, thus enhancing their psychological safety (Higgins, 1998). Furthermore, promotion-oriented employees are generally more open to the uncertainties that may arise in their work and are more willing to embrace the challenges introduced by customer empowering behavior. As a result, they experience stronger intrinsic motivation to respond positively to customer empowering behavior, which further strengthens their psychological safety (Gao et al., 2020). Additionally, promotion-focused employees are more attuned to positive cues in their environment. When they perceive customer empowering behavior, they are more likely to interpret it as an expression of trust in their abilities, which fosters greater psychological safety. Therefore, this study proposes:

*H3a*: Promotion focus moderates the relationship between customer empowering behavior and psychological safety. For employees with higher promotion focus, the positive effect of customer empowering behavior on psychological safety is stronger.

At the same time, accepting customer empowering behavior also entails greater work responsibilities and pressure (Higgins, 1997). As a result, not every employee is willing to accept increased empowerment from customers. For frontline service employees with a prevention focus, who are more concerned with avoiding negative consequences and losses, the potential risks of customer empowering behavior are particularly salient. These employees are likely to focus on the potential adverse effects of customer empowerment, adopting conservative strategies to minimize any risks (Higgins, 1998; Zou and Chan, 2019). Rather than viewing customer empowering behavior as an opportunity for learning, self-improvement, or achieving work and career goals, prevention-focused employees are more concerned with how the autonomy granted by customers might jeopardize their service delivery and result in negative outcomes if they fail to perform their tasks well (Gao et al., 2020). Even if they perceive customer empowering behavior, they may fear that independent decision-making could lead to customer dissatisfaction and, consequently, personal or professional losses. This fear can make them adopt a cautious, conservative approach, leading to a reduced sense of psychological safety. As a result, for employees with a prevention focus, the positive impact of customer empowering behavior on psychological safety is significantly diminished. Therefore, this study proposes:

*H3b*: Prevention focus moderates the relationship between customer empowering behavior and psychological safety. For employees with higher levels of prevention focus, the positive effect of customer empowering behavior on psychological safety is weaker.

Based on the reasoning behind the aforementioned mediation and moderation hypotheses, we conclude that employee psychological safety mediates the relationship between customer empowering behavior and proactive service behaviors. The strength of this effect is moderated by employees’ regulatory focus. Specifically, for employees with a promotion focus, psychological safety enhances proactive customer service behaviors. In contrast, frontline service employees with a prevention focus, when perceiving customer empowering behavior, focus more on potential negative consequences. This focus does not significantly improve their psychological safety or further enhance their proactive service behaviors. Therefore, we hypothesize that both promotion and prevention focus significantly moderate the mediating effect of psychological safety. Specifically, the indirect effect of customer empowering behavior on proactive service behaviors through psychological safety is moderated by employees’ promotion and prevention focus. Consequently, this study proposes:

*H4a*: Promotion focus moderates the indirect effect of customer empowering behavior on employee proactive customer services through psychological safety. Specifically, when employees have a higher level of promotion focus, the positive effect of customer empowering behavior on employee proactive customer services through psychological safety is stronger.

*H4b*: Prevention focus moderates the indirect effect of customer empowering behavior on employee proactive customer services through psychological safety. Specifically, when employees have a higher level of prevention focus, the positive effect of customer empowering behavior on employee proactive customer services through psychological safety is weaker.

**Figure 1:**
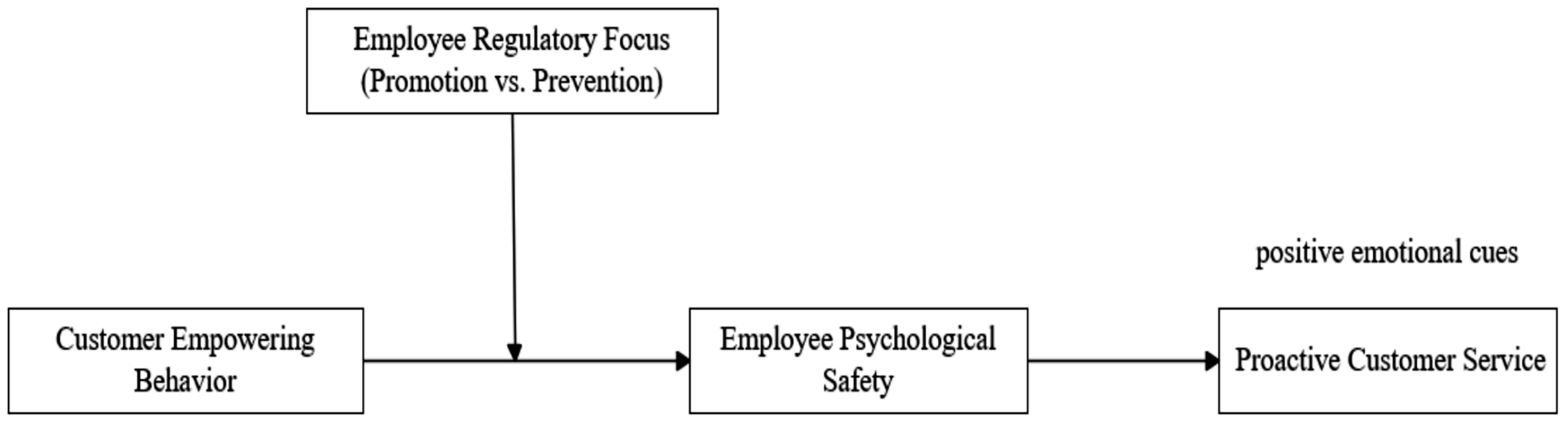
Research framework.

The research framework of this paper is shown in Figure 1.

### Study design

1 Research subjects and data collection process.

The data for this study were collected from frontline service employees and their immediate supervisors in typical service enterprises located in Bangkok and Pattaya, Thailand, including 10 restaurants and 10 massage parlors. These organizations were selected because their service delivery processes require frequent and direct interactions between customers and employees, making them particularly suitable for the research context. To mitigate potential common method bias (Podsakoff et al., 2012), the study employed a multi-source, multi-stage, time-lagged survey design. Data were collected in three waves, each spaced 1 month apart. Specifically, frontline employees completed self-report questionnaires, while their direct supervisors evaluated employee behaviors, enabling a more objective assessment of the outcome variables. Prior to data collection, with assistance from the human resources managers or store managers, a complete list of eligible participants was obtained. All participants were assigned unique identification codes to ensure accurate matching of responses across different survey waves and sources. This design enhanced the internal validity of the study and helped ensure the reliability of the data collected.

In the first phase of the survey, employees self-reported their perceived customer-empowering behavior, regulatory focus, and demographic information. The second phase focused on measuring employees’ psychological safety. In the third phase, direct supervisors of the participating employees assessed their proactive customer service behaviors. A total of 398 questionnaires were distributed in the initial survey, with 309 valid responses received, yielding a valid response rate of 77.64%. The second survey was conducted exclusively with these 309 employees, resulting in 258 valid responses and a valid response rate of 83.50%. In the final phase, questionnaires were sent to the direct supervisors of the 258 employees who had completed the second survey. A total of 33 supervisors evaluated the proactive customer service behaviors of these employees, producing 236 valid supervisor–subordinate matched responses (from 32 supervisors), yielding a valid response rate of 91.47%.

Among the valid samples, 132 were employees from restaurants, and 104 were from massage parlors. The sample was predominantly male, accounting for 62.3% of respondents. Age distribution was relatively balanced, with 18.2% of participants under 20 years old, 33.1% between 21 and 30 years old, 16.9% between 31 and 40 years old, 12.7% between 41 and 50 years old, and 19.1% over 51 years old. Regarding educational background, the majority of respondents had completed high school or lower (69.5%), followed by those with technical secondary school education (20.3%), and the smallest proportion had a college or undergraduate degree (10.2%). As for work experience, 32.2% had been employed for 1–2 years, 38.1% for 3–4 years, and 29.7% for 5 years or more.

2 Measurement instruments.

Since the measurement instruments used in this study were adapted from established, high-quality English-language scales published in leading international journals, the translation and back-translation of the items were meticulously conducted by three field experts and five doctoral students in management. To ensure the questionnaire’s relevance to the service industry, eight frontline service employees from restaurants and massage parlors were recruited to review the items for clarity and appropriateness. These employees were excluded from the subsequent formal survey to avoid duplication in the sample. Revisions were made to the wording of the items as necessary, while preserving the original meaning. This study utilized a Likert 5-point scale for the responses.

Customer empowering behavior was measured using the 8-item scale developed by Dong et al. (2015), which includes items such as, “The customer allows me to make decisions about how to meet his/her needs.” The Cronbach’s *α* coefficient for this scale in the current study was 0.82.

Employee psychological safety was assessed using the 5-item scale developed by Liang et al. (2012), which includes items such as, “In my workplace, I can express my ideas freely.” The Cronbach’s α coefficient for this scale in the present study was 0.76.

Employee regulatory focus was measured using the 11-item scale developed by Higgins et al. (2001), which includes two dimensions: promotion focus and prevention focus. The promotion focus dimension consists of 6 items, such as, “I often perform well when trying different things.” The Cronbach’s α coefficient for this dimension in the current study was 0.76. The prevention focus dimension includes 5 items, such as, “Being careful and cautious often helps me avoid trouble.” The Cronbach’s α coefficient for this dimension was 0.89 in the present study.

Employees’ proactive service behavior was measured using the 7-item scale developed by Rank et al. (2007), which includes items such as, “The employee actively seeks customer feedback to ensure that customer expectations are met.” The Cronbach’s α coefficient for this scale in the present study was 0.84.

Based on the findings of previous studies (e.g., Author, Year), this study included employee demographic information and industry type as control variables to strengthen the inference of causal relationships between the key variables.

3 Data analysis strategy.

All statistical analyses were conducted using SPSS 26.0 (with the PROCESS macro) and AMOS 24.0. Specifically, SPSS 26.0 was used to perform descriptive statistics, correlation analyses, hierarchical regression analyses, interaction tests, and bootstrapping analyses for mediation and moderated mediation effects. Following the procedures recommended by Aiken and West (1991), all predictor and moderator variables were mean-centered prior to creating interaction terms.

Confirmatory factor analyses (CFA) and common method bias tests were conducted using AMOS 24.0. Model fit was evaluated using multiple indices, including the chi-square statistic (χ^2^), root mean square error of approximation (RMSEA), comparative fit index (CFI), and incremental fit index (IFI).

### Data analysis results

1 Common method bias test.

Since most variables in the model were measured using employee self-reports, and the independent and moderator variables were collected at the same time point, the validity of the research conclusions could potentially be influenced by common method bias. To test for this bias, Harman’s single-factor test was conducted. The results indicated that the variance explained by the first unrotated factor was 17.18%, which is well below the critical threshold of 40%, suggesting that common method bias was not a major concern. Additionally, a more rigorous test was conducted by controlling for an unmeasured latent method factor, as recommended by Podsakoff et al. (2012). The model comparison results (see Table 1) showed that changes in RMSEA, CFI, and IFI were all less than 0.01, further indicating that common method bias was not a serious issue (Tu et al., 2014).

1 Confirmatory factor analysis.

**Table 1:** Confirmatory factor analysis.

Model	*χ* ^2^	df	*χ*^2^/df	IFI	CFI	RMSEA
Five-factor model with common bias	484.12	288	1.68	0.90	0.90	0.05
Five-factor model	491.02	289	1.70	0.90	0.90	0.06
Four-factor model^a^	620.61	293	2.12	0.84	0.84	0.07
Four-factor model^b^	625.01	293	2.13	0.84	0.84	0.07
Four-factor model^c^	725.16	295	2.48	0.79	0.79	0.07
Four-factor model^d^	726.88	295	2.46	0.79	0.79	0.07
Single-factor model^e^	1,726.14	296	5.84	0.62	0.62	0.14

*N* = 236. ^a^Customer empowering behavior and psychological safety combined; ^b^facilitative and defensive regulation combined; ^c^psychological safety and employee proactive customer service combined; ^d^customer empowering behavior and employee proactive customer service combined; ^e^all variables combined.

The results of the confirmatory factor analysis (see Table 1) indicated that the five-factor model demonstrated a good fit to the data: *χ*^2^(289) = 491.02, RMSEA = 0.06, IFI = 0.90, CFI = 0.90. Additionally, all factor loadings ranged from 0.56 to 0.87, suggesting acceptable convergent validity and strong discriminant validity among the five key constructs.

3 Descriptive statistical analysis.

The results of the descriptive statistical analysis are presented in Table 2. Positive correlations were observed among the independent variables, mediating variable, and outcome variable. These results are consistent with the proposed hypotheses and provide a preliminary basis for further hypothesis testing.

**Figure 2:**
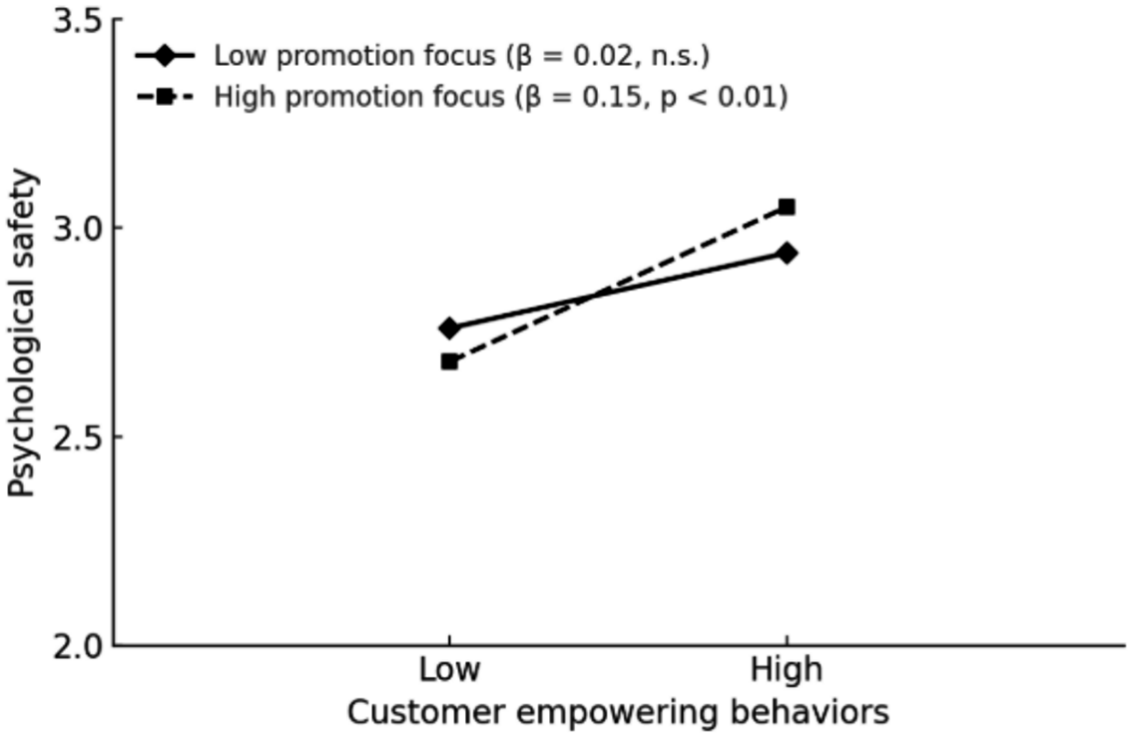
Interactive effects of promotion focus.

**Figure 3:**
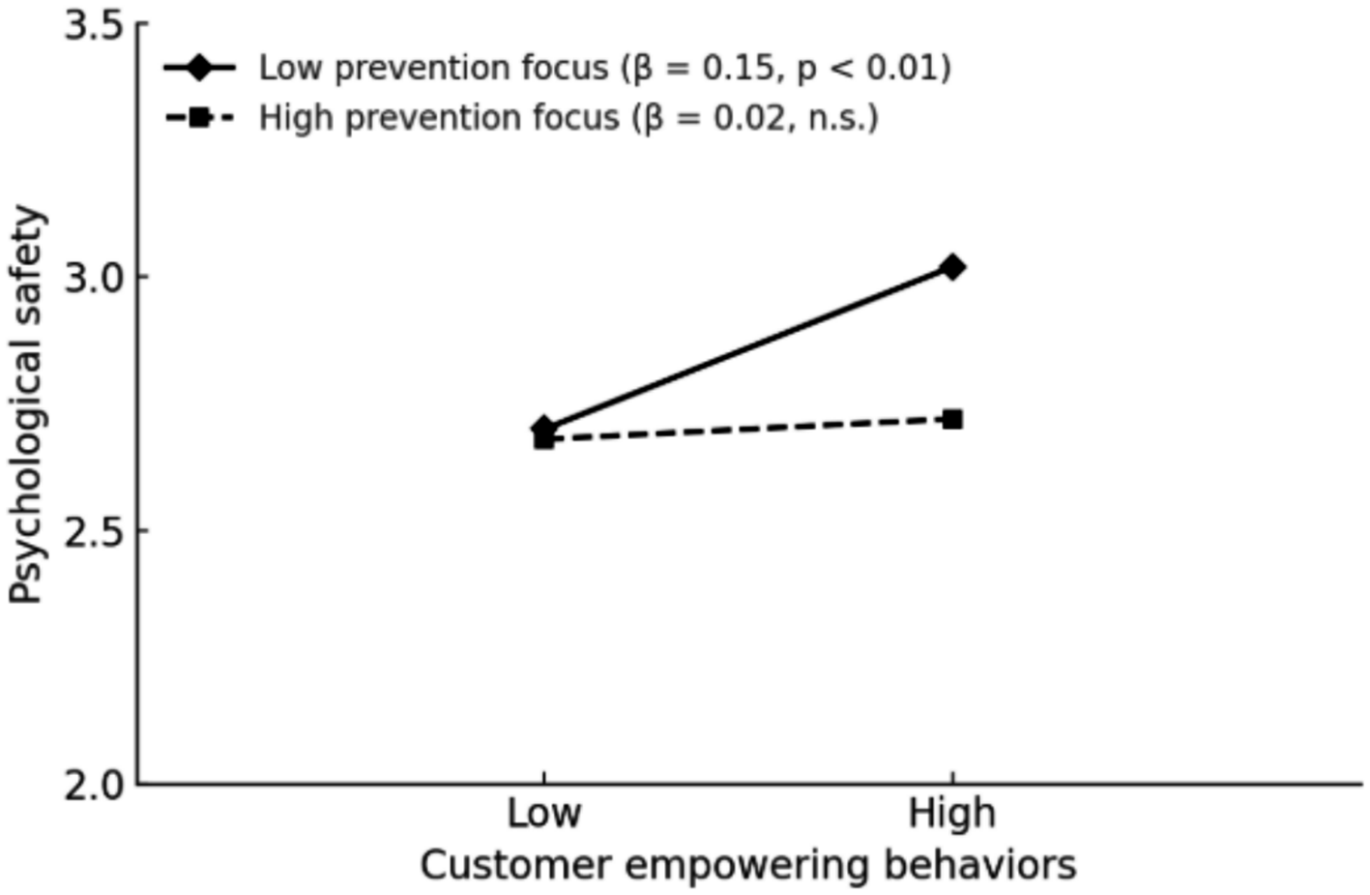
Interaction effect of prevention focus.

4 Hypothesis testing.
5 As shown in Model 6 (M6) of Table 3, customer empowering behavior had a significant positive effect on employee proactive customer service behavior (*β* = 0.33, *p* < 0.01), thus supporting Hypothesis 1 (H1). Psychological safety also had a significant positive effect on proactive customer service behavior (M7: *β* = 0.25, *p* < 0.01). After introducing psychological safety as a mediating variable, the effect remained significant (M8: *β* = 0.17, *p* < 0.01), while the direct effect of customer empowering behavior decreased but remained statistically significant (M8: *β* = 0.29, *p* < 0.01). These results indicate that psychological safety partially mediates the relationship between customer empowering behavior and proactive customer service behavior, providing partial support for Hypothesis 2 (H2). Furthermore, the indirect effect of customer empowering behavior on proactive customer service behavior through psychological safety was 0.06, with a 95% confidence interval of [0.01, 0.12], excluding zero, which further supports H2.
6 To examine the moderating effects, this study first standardized the variables for customer empowering behavior, promotion focus, and prevention focus. Two interaction terms were then created: customer empowering behavior × promotion focus and customer empowering behavior × prevention focus. As shown in Model 4 (M4) of Table 3, the interaction between customer empowering behavior and promotion focus had a significant positive effect on psychological safety (*β* = 0.13, *p* < 0.05), while the interaction between customer empowering behavior and prevention focus had a significant negative effect on psychological safety (*β* = −0.14, *p* < 0.05). These results provide support for Hypotheses 3a (H3a) and 3b (H3b). In accordance with the recommendations of Aiken and West (1991), interaction effects were plotted to aid interpretation (see Figures 2 and 3).
7 As shown in Table 4, the indirect effect of customer empowering behavior on employee proactive customer service behavior was stronger when promotion focus was high (*β* = 0.10, 95% CI [0.02, 0.23]), but weaker when promotion focus was low (*β* = 0.00, 95% CI [−0.04, 0.04]). The difference between the two groups was statistically significant (*β* = 0.09, 95% CI [0.01, 0.25]), providing support for Hypothesis 4a (H4a). Similarly, when prevention focus was low, the indirect effect of customer empowering behavior on employee proactive customer service behavior was stronger (*β* = 0.10, 95% CI [0.02, 0.20]), but weaker when prevention focus was high (*β* = 0.01, 95% CI [−0.03, 0.04]). The between-group difference was also significant (*β* = −0.09, 95% CI [−0.19, −0.01]), thereby supporting Hypothesis 4b (H4b).

**Table 2:** Mean, standard deviation and correlation coefficient of each variable.

Variable	1	2	3	4	5	6	7	8	9	10
1. Gender										
2. Age	0.15^*^									
3. Education level	−0.01	0.01								
4. Age group	0.03	0.48^**^	0.04							
5. Occupation	−0.06	0.01	0.02	0.26^**^						
6. Customer empowering behavior	−0.06	−0.11	−0.06	−0.11^**^	−0.03					
7. Promotion focus	0.03	−0.07	−0.06	−0.10	0.09	−0.11^**^				
8. Prevention focus	−0.06	−0.17^**^	0.17^**^	0.07	−0.26^**^	0.26^**^	−0.25^**^			
9. Psychological safety	0.17^**^	0.26^**^	−0.11^**^	−0.07	0.09	0.11^**^	−0.12^**^	0.76^**^		
10. Employee service proactive behavior	−0.09	0.17^**^	0.32^**^	0.07	0.13^**^	0.25^**^	−0.20^**^	0.76^**^	0.79^**^	
Mean (M)	1.62	1.84	1.61	1.84	1.68	1.62	3.44	3.67	3.72	3.75
Standard deviation (SD)	0.49	0.39	0.43	0.50	0.45	0.43	1.13	1.44	1.13	1.21

*N* = 236; ^∗^ indicates *p* < 0.05, ^∗∗^ indicates *p* < 0.01; the values in brackets are the *α* coefficients for each scale.

**Table 3:** Hierarchical regression results.

Variable	Psychological safety				Proactive customer service			
	M1	M2	M3	M4	M5	M6	M7	M8
Control variables								
Gender	−0.07	−0.05	−0.03	−0.02	−0.01	0.02	0.01	−0.01
Age	0.16^*^	0.14	0.18	0.15	0.12	0.03	−0.06	−0.03
Education level	−0.19^**^	−0.13^**^	−0.17^**^	−0.17^**^	−0.07	−0.06	−0.09	−0.03
Job tenure	0.08	0.05	0.06	0.05	−0.09	−0.09	−0.11	−0.13^*^
Industry	0.08	0.08	0.06	0.05	−0.09	−0.11	−0.12	−0.13^*^
Independent variables								
Customer empowering behavior		0.24^**^	0.26^**^	0.19^**^		0.33^**^		0.29^**^
Mediator variables								
Psychological safety							0.25^**^	0.17^**^
Moderator variables								
Promotion focus			0.02	0.04				
Prevention orientation			−0.17^**^	−0.17^**^				
Interaction terms								
Customer empowering behavior × Promotion focus				0.13^*^				
Customer empowering behavior × Prevention orientation				−0.14^*^				
Model fit statistics								
*F*	3.75	5.0.84	5.57	5.96	1.15	5.73	3.38	6.06
Δ*F*	3.75	3.75	3.75	6.43^*^	6.43^**^	1.15	27.92^**^	6.20^**^
*R* ^2^	0.08	0.09	0.16	0.21	0.13	0.11	0.16	0.16
Δ*R*^2^	0.08^*^	0.05^**^	0.03^*^	0.05^**^	0.11^**^	0.06^**^	0.03^**^	0.03^**^

*N* = 236; ^∗^ indicates *p* < 0.05, ^∗∗^ indicates *p* < 0.01.

**Table 4:** Analysis of moderated mediation effects.

Moderator variable	Customer empowering behavior → Psychological safety → Employee proactive customer service			
	Effect value	Boot SD	95% LLCI	95% ULCI
Promotion focus				
Mean −1 Standard Deviation	0.00	0.02	−0.04	0.04
Mean	0.05	0.03	0.01	0.12
Mean +1 Standard Deviation	0.10	0.05	0.02	0.23
Between-group differences	0.09	0.06	0.01	0.25
Prevention focus				
Mean −1 Standard Deviation	0.10	0.05	0.02	0.25
Mean	0.05	0.03	0.01	0.11
Mean +1 Standard Deviation	0.01	0.02	0.02	0.04
Between-group differences	−0.09	0.05	−0.19	−0.01

*N* = 236; Bootstrap samples = 5,000.

## Conclusion and discussion

1 Research findings.

This study adopted a multi-source, multi-wave, time-lagged research design involving 236 frontline service employees and their 32 direct supervisors in the service industry. The research aimed to examine the underlying mechanisms and boundary conditions through which customer empowering behavior influences employee proactive customer service. The key findings of the study are as follows:

(1) Customer empowering behavior exerts a positive influence on employees’ proactive service behavior, and this relationship is mediated by psychological safety. Although customer empowering behavior grants frontline employees a certain degree of autonomy in decision-making, its successful translation into proactive service behavior depends on whether employees experience a clear sense of psychological safety during the process (Detert and Burris, 2007). Only when employees are confident that their psychological safety is sufficiently high are they willing to engage in more proactive service behaviors, in accordance with the principle of social exchange.
(2) A promotion focus strengthens the positive relationship between customer empowering behavior and employee psychological safety, whereas a prevention focus weakens this relationship. This finding suggests that, during service encounters, although customer empowering behavior conveys trust and affirmation from customers and grants employees a degree of autonomy, increased empowerment also entails greater responsibility. Not all employees are equally willing to accept such responsibility. In these circumstances, employees’ regulatory focus becomes a critical moderating factor. Specifically, a promotion focus (as opposed to a prevention focus) amplifies the positive impact of customer empowering behavior on psychological safety (Zou and Chan, 2019).
(3) The regulatory role of regulatory focus in shaping the outcomes of customer empowering behavior also manifests as a moderated mediation effect. Specifically, when employees with a promotion focus perceive customer empowering behavior, they tend to focus on potential positive outcomes (Higgins, 1997), which enhances their psychological safety and, in turn, promotes more customer-oriented proactive customer services. In contrast, employees with a prevention focus are more attuned to possible negative consequences (Higgins, 1998), which impedes the development of psychological safety and consequently weakens the likelihood of proactive service behavior.

2 Theoretical contribution.

(1) This study centers on the relatively novel concept of customer empowering behavior (CEB) and extends its influence to employees’ proactive service behavior, thereby enriching current understanding of the downstream effects of CEB. By investigating the impact of CEB on employee proactivity, this study identifies an important antecedent of proactive service behavior and contributes new theoretical perspectives to research on the factors influencing such behaviors. Although a few existing studies have preliminarily explored the positive effects of CEB on employees’ current service performance (Dong et al., 2015; Tuan et al., 2019) and long-term career growth (Guo and Cheng, 2021), the question of whether—and how—CEB promotes proactive service behavior remains underexplored. In real-world service encounters, frontline employees are frequently required to address urgent and dynamic customer needs, necessitating both flexibility and initiative (Griffin et al., 2007). In such contexts, proactive service behavior becomes a critical driver of high-quality service outcomes (Zhu et al., 2017). The current findings reveal that CEB serves as a key motivational resource that encourages employees to engage in proactive service behaviors. These insights offer valuable theoretical implications by advancing the literature on both CEB and proactive service behavior, and lay the foundation for future research in this domain.
(2) Grounded in social exchange theory, this study uncovers the mediating role of psychological safety in the relationship between customer empowering behavior (CEB) and employee proactive service behavior. By doing so, it opens the “black box” of how CEB translates into employee action, offering a critical theoretical foundation for understanding how and why CEB fosters proactive service behaviors among frontline employees. While prior studies have acknowledged the positive impact of CEB on employee attitudes and performance, few have investigated the underlying mechanisms. This study fills that gap by incorporating psychological safety as a central mediating variable and empirically validating its role. Specifically, the results demonstrate that when employees perceive CEB—i.e., when customers affirm their competence and grant them autonomy—it enhances their psychological safety, which in turn stimulates greater proactive engagement in service delivery. Moreover, by situating the analysis within Chinese service industry settings, this research provides a culturally contextualized validation of social exchange theory. In doing so, it highlights the universality and adaptability of this theoretical lens across diverse cultural contexts. The findings extend the practical explanatory power of social exchange theory, particularly in dynamic and interpersonal environments such as service encounters (Xi et al., 2018).
(3) Drawing on social exchange theory and integrating insights from regulatory focus theory, this study examines the boundary conditions under which customer empowering behavior (CEB) exerts differential effects on employees. Although CEB grants employees autonomy, it also increases their responsibility (Cheong et al., 2016; Podsakoff et al., 2012). As such, not all employees are equally receptive to being empowered by customers. The findings reveal that employees’ regulatory focus—specifically, promotion focus versus prevention focus—plays a pivotal moderating role in shaping the effects of CEB. Employees with a promotion focus, who are oriented toward growth, advancement, and potential gains, tend to interpret CEB as a motivating and affirming cue. In contrast, employees with a prevention focus, who are more concerned with security, risk avoidance, and potential losses, may perceive CEB as pressure-laden or even threatening. These results offer a nuanced understanding of when and for whom customer empowering behavior is most effective, and underscore the importance of considering individual differences in motivational orientation when evaluating the outcomes of customer empowerment. This contributes to a more precise theoretical framework for understanding the interplay between external empowerment cues and internal regulatory processes.

3 Practical implications.

(1) Given the widespread presence of customer empowering behavior (CEB) in the service sector and the strategic importance of employees’ proactive customer service behavior in fostering customer trust and loyalty (Guo and Cheng, 2021; Zhu et al., 2017), it is essential for both managers and frontline service staff to enhance their awareness and understanding of CEB. This study identifies psychological safety as the key mediating mechanism linking CEB to proactive service behavior. Specifically, CEB first enhances employees’ psychological safety, which then encourages them to engage in more proactive, customer-oriented behaviors. This finding underscores the importance of fostering a psychologically safe work environment in which employees feel confident and supported in making autonomous service decisions. From a managerial perspective, organizations should intentionally enhance employee autonomy and simultaneously reduce the psychological risks associated with acting independently. Managers can accomplish this by reinforcing trust, encouraging decision-making latitude, and creating an error-tolerant service culture. Such efforts can increase employees’ subjective willingness and objective capability to respond positively to CEB, ultimately promoting better service outcomes and enabling service firms to generate greater customer value.
(2) This study reveals that the impact of customer empowering behavior (CEB) varies according to employees’ regulatory focus. Specifically, a promotion focus amplifies the positive effect of CEB on proactive service behavior, while a prevention focus weakens it. These findings offer meaningful guidance for service-oriented organizational management. On one hand, managers should recognize and leverage the strengthening effect of a promotion focus. Employees with a promotion orientation are more responsive to the autonomy and trust embedded in CEB, translating it into greater psychological safety and proactive behavior. Therefore, managers are encouraged to cultivate a work environment that reinforces this orientation—for example, by actively soliciting employee feedback, fostering positive leader–employee communication, and creating opportunities for goal-oriented development. These practices can enhance psychological safety and promote the internalization of CEB as a motivating resource. On the other hand, managers must also acknowledge the inhibiting role of a prevention focus. Employees with a prevention orientation may perceive customer empowerment as pressure or risk, which can undermine their sense of security and reduce their proactive engagement. To mitigate this, organizations should offer structural and emotional support—such as reinforcing a psychologically safe climate, normalizing controlled risk-taking, and signaling tolerance for mistakes. These strategies can help employees feel protected even in autonomous roles, thereby reducing the psychological resistance caused by a prevention focus and enabling them to respond more positively to customer empowerment cues.

4 Research limitations and prospects.

This study also has some shortcomings:

(1) One notable limitation of this study is that most variables were measured through employee self-reports, which may compromise the ability to draw robust causal inferences. Self-assessment methods are inherently subject to common method bias and perceptual distortion, thereby limiting the internal validity of the findings. To enhance the scientific rigor and reliability of future research, scholars are encouraged to adopt more rigorous research designs, such as experimental or longitudinal studies, which can better establish causality. In addition, employing multi-source data collection—for instance, gathering employee proactive service behavior ratings from customers or supervisors—can help reduce common method variance and improve measurement accuracy. Such methodological improvements would contribute to a more nuanced and objective understanding of the mechanisms and outcomes associated with customer empowering behavior.
(2) This study confirmed the partial mediating role of psychological safety in the relationship between customer empowering behavior (CEB) and employees’ proactive customer service behavior. This finding suggests that additional mediating mechanisms may exist in this process that have not yet been fully explored. Future research could further investigate the psychological and behavioral consequences of CEB by incorporating alternative theoretical frameworks such as role theory and self-consistency theory. These perspectives may provide valuable insights into how employees interpret and internalize empowering cues from customers, and how such interpretations affect their workplace behaviors and attitudes. In addition, future studies could expand the scope of outcome variables beyond proactive service behavior to include more diverse and holistic constructs, such as job satisfaction, emotional well-being, or even family-related outcomes like work–family enrichment or family happiness. A broader examination of outcomes will contribute to a more comprehensive understanding of the multi-dimensional effects of CEB on employees’ professional and personal lives.
(3) Grounded in regulatory focus theory, this study verified the differential moderating effects of promotion focus and prevention focus on the relationship between customer empowering behavior (CEB) and employee outcomes. While these findings contribute to the expansion of existing literature, there remains considerable room for further exploration. Future research could investigate additional boundary conditions that may influence the effectiveness of CEB. For example, from a team-level perspective, examining the moderating role of leadership support may provide deeper insight into how contextual factors shape employees’ responses to empowerment from customers. From an organizational-level perspective, future studies might explore whether transformational leadership strengthens or buffers the effects of CEB by fostering an environment that encourages autonomy, innovation, and risk-taking. These directions would offer a more comprehensive understanding of the multilevel contingencies under which CEB influences employee attitudes and behaviors.

## Data Availability

The original contributions presented in the study are included in the article/supplementary material, further inquiries can be directed to the corresponding author.
